# Systematic Review and Meta‐Analysis on the Efficacy and Safety of Salvage Esophagectomy for T4 Esophageal Squamous Cell Carcinoma

**DOI:** 10.1002/ags3.70233

**Published:** 2026-05-05

**Authors:** Makoto Sakai, Kengo Kuriyama, Kazue Nagai, Ken Shirabe, Hiroshi Saeki

**Affiliations:** ^1^ Division of Gastroenterological Surgery, Department of General Surgical Science Gunma University, Graduate School of Medicine Maebashi Japan; ^2^ Gunma University Center for Food Science and Wellness Maebashi Japan; ^3^ Innovative Medical Research Center Gunma University Hospital Maebashi Japan; ^4^ Department of General Surgical Science Gunma University, Graduate School of Medicine Maebashi Japan

**Keywords:** esophageal cancer, salvage esophagectomy, T4

## Abstract

**Background:**

Salvage esophagectomy is associated with high morbidity and mortality rates. We performed a systematic review and meta‐analysis to evaluate the efficacy and safety of salvage esophagectomy for unresectable locally advanced (T4) esophageal squamous cell carcinoma (ESCC).

**Methods:**

We searched the MEDLINE (PubMed) databases, the Cochrane Library databases, Ichushi‐Web (the databases of the Japan Medical Abstract Society), and CiNii (the Academic Information Search Service of the National Institute of Information from Japan) for articles on salvage esophagectomy for T4 ESCC.

**Results:**

Eight studies (208 cases) were eligible for meta‐analysis. The overall postoperative complications rate (Clavien–Dindo grade ≥ III) was 30% (95% CI: 23–38, *I* ^2^ = 0%); anastomotic leak rate was 18% (95% CI: 13–25, *I* ^2^ = 15%); pulmonary complication rate was 31% (95% CI: 20–42, *I* ^2^ = 63%); mortality rate was 7% (95% CI: 3–11, *I* ^2^ = 0%); and R0 resection rate was 72% (95% CI: 59–83, *I* ^2^ = 72%). A meta‐analysis of survival was not performed due to heterogeneous reporting across studies. However, overall survival was reported to be higher in patients achieving R0 resection.

**Conclusions:**

Salvage esophagectomy for cT4 ESCC is associated with significant morbidity, particularly a high anastomotic leak rate of 18%. The procedure should be reserved for carefully selected patients in whom R0 resection is highly feasible.

## Graphical abstract

This meta‐analysis of 208 cases shows that salvage esophagectomy for cT4 esophageal squamous cell carcinoma achieves a 72% R0 resection rate, offering a curative pathway for selected patients. However, it remains a high‐risk procedure with an 18% anastomotic leak rate and 30% major complications (Clavien–Dindo ≥ III). Stringent patient selection and specialized management are essential to balance oncological benefits against surgical risks.

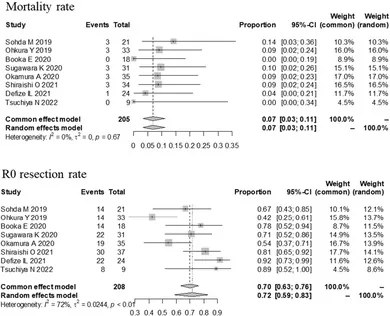

## 1 Introduction

Esophageal cancer (EC) is the seventh leading cause of cancer‐related death and the 11th most common cancer worldwide [1]. The 10‐year net survival rate of EC diagnosed in 2011 was approximately 30% in Japan [2]. Concurrent chemoradiotherapy (CRT) is the standard therapy for unresectable locally advanced (T4) esophageal squamous cell carcinoma (ESCC) [3]. However, the curability of CRT for T4 ESCC remains limited with respect to local control and survival rates [4, 5, 6, 7]. Salvage esophagectomy is currently considered the only established therapeutic strategy that offers possible long‐term survival for patients with ESCC experiencing local failure after definitive CRT (dCRT) [8, 9].

Previous studies have reported that salvage esophagectomy is associated with high morbidity and mortality rates [8, 10]. On the other hand, the benefits of this high‐risk surgery for patients with T4 ESCC who become resectable following definitive CRT remain unclear because of the limited number of applicable cases. In this study, we conducted a meta‐analysis to systematically evaluate the efficacy and safety of salvage esophagectomy for T4 ESCC in this limited patient cohort.

## 2 Methods

### 2.1 Literature Search

We conducted a meta‐analysis data search following the PRISMA statement guidelines [11]. A comprehensive computerized literature search was conducted using the MEDLINE database (PubMed), the Cochrane Library databases, Ichushi‐Web (the databases of the Japan Medical Abstract Society), and CiNii (the Academic Information Search Service of the National Institute of Information and Communications Technology in Japan). We searched PubMed for relevant studies with the following Medical Subject Heading (MeSH) terms and text words: (“oesophageal cancer”[All Fields] OR “esophageal neoplasms”[MeSH Terms] OR (“esophageal”[All Fields] AND “neoplasms”[All Fields]) OR “esophageal neoplasms”[All Fields] OR (“esophageal”[All Fields] AND “cancer”[All Fields]) OR “esophageal cancer”[All Fields]) AND (“unresectability”[All Fields] OR “unresectable”[All Fields] OR “unresected”[All Fields] OR ((“focal”[All Fields] OR “focalities”[All Fields] OR “focality”[All Fields] OR “focalization”[All Fields] OR “focalized”[All Fields] OR “focally”[All Fields] OR “focals”[All Fields] OR “local”[All Fields] OR “localisation”[All Fields] OR “localisations”[All Fields] OR “localise”[All Fields] OR “localised”[All Fields] OR “localises”[All Fields] OR “localising”[All Fields] OR “localization”[All Fields] OR “localizations”[All Fields] OR “localize”[All Fields] OR “localized”[All Fields] OR “localizer”[All Fields] OR “localizers”[All Fields] OR “localizes”[All Fields] OR “localizing”[All Fields] OR “locally”[All Fields] OR “locals”[All Fields]) AND (“advance”[All Fields] OR “advanced”[All Fields] OR “advancement”[All Fields] OR “advancements”[All Fields] OR “advances”[All Fields] OR “advancing”[All Fields])) OR (“T4b”[All Fields] OR “T4”[All Fields])) AND (“salvage”[All Fields] OR “salvageability”[All Fields] OR “salvageable”[All Fields] OR “salvaged”[All Fields] OR “salvages”[All Fields] OR “salvaging”[All Fields]). We searched the Cochrane Library databases using the following search terms: (((esophageal cancer) AND (((unresectable) OR (locally advanced)) OR ((T4b) OR (T4)))) AND (salvage)): ti, ab,kw. We also searched Ichushi‐Web using the following search terms in Japanese: (((Esophageal neoplasms [TH] OR Esophageal cancer [AL])) AND (Locally advanced [AL]) AND ((Chemoradiotherapy [TH] OR Chemoradiation therapy [AL])) AND ((Salvage therapy [TH] OR Salvage surgery [AL]))). We searched CiNii using the following search terms in Japanese: “esophageal cancer”, “salvage”, and “T4”.

### 2.2 Study Selection

We included studies that met the following criteria: (1) were designed as observational studies, multicenter reports, single‐center series, case reports, or case–control studies; (2) reported salvage esophagectomy for initially unresectable locally advanced ESCC after chemoradiation with a total dose of 50.4–60Gy; and (3) reported postoperative complication rate, mortality rate; and R0 resection rate. We excluded case reports and case series with fewer than three participants to reduce inclusion bias.

### 2.3 Data Extraction and Quality Evaluation

The following information was extracted as endpoints separately for each procedure: overall postoperative complication rate, anastomotic leak rate, pulmonary complication rate, mortality rate, and R0 resection rate. Definition of postoperative mortality varied across the included studies (e.g., 30‐day, 90‐day, in‐hospital, or surgery‐related mortality). Despite these variations in terminology and timeframes, we performed the analysis on the premise that all studies fundamentally aimed to capture deaths directly attributable to the surgical intervention or the subsequent hospital stay. The quality of each included study was assessed by two researchers (MS and KK) using the Joanna Briggs Institute (JBI) Critical Appraisal Checklist for Case Series [12]. Any disagreements among the authors were resolved through discussion. The evidence was rated based on the guidelines set by the Oxford Centre for Evidence‐based Medicine [13], ranging from level 1 (strongest) to level 5 (weakest).

### 2.4 Statistical Analysis

All statistical analyses were performed using EZR [14]. The metaprop command (meta‐analysis of single proportions) was used to find the pooled estimate of endpoints and corresponding confidence intervals. The Freeman–Tukey double arcsine transformation was used to stabilize the variance of individual study estimates. Each study was weighed in both common‐ and random‐effects models. A *p*‐value < 0.05 indicated a significant difference. Heterogeneity among studies was assessed using the *I* ^2^ statistic, and *p* < 0.10 was considered significant. The DerSimonian–Laird estimator was used to calculate the heterogeneity variance, *τ* ^2^ [15]. In cases of significant heterogeneity among studies, a sensitivity analysis was conducted by sequentially omitting each study to assess its impact on the pooled results. Due to the limited number of studies included, Doi plot and Luis Furuya–Kanamori (LFK) index were used to determine the presence of publication biases related to each outcome. On the Doi plot, a symmetrical, mountain‐like graph with LFK index values within ±1 indicates no asymmetry; values between ±1 and ±2 suggest minor asymmetry; and values exceeding ±2 indicate major asymmetry [16]. The statistical methods were reviewed by a biostatistician (KN).

## 3 Results

### 3.1 Literature Search and Study Selection

**FIGURE 1:**
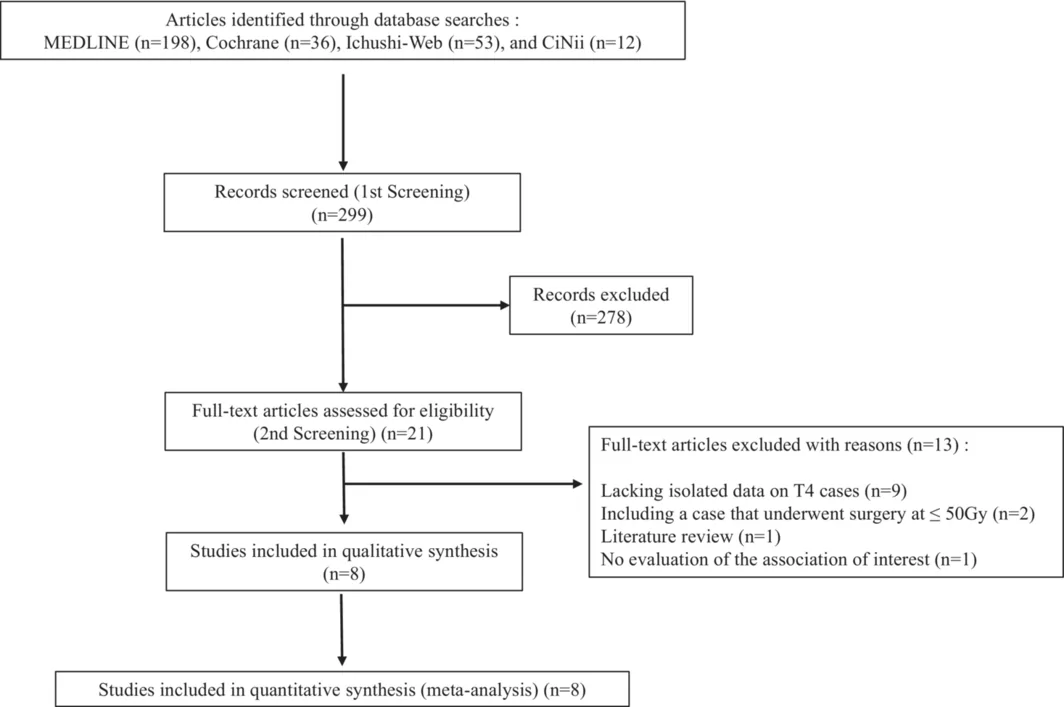
Flow diagram of the systematic literature search.

A total of 299 articles were identified through a search of MEDLINE, the Cochrane Library databases, Ichushi‐Web, and CiNii. Of these, 278 were excluded after screening titles and abstracts, and 21 studies were considered potentially valuable and assessed for eligibility. Eight studies were considered eligible for meta‐analysis. A flow chart of the search process is shown in Figure 1.

### 3.2 Characteristics of the Selected Studies

The characteristics of the included studies are summarized in Table 1. All included studies [17, 18, 19, 20, 21, 22, 23, 24] were case series; thus, the evidence level was considered level 4 (the second weakest). The studies were published between 2019 and 2022 and included 208 cases. Seven studies were conducted in Japan and one in the Netherlands. Although one study [23] included a single case of adenocarcinoma, we judged its impact on the overall results to be minimal and therefore included the study in the analysis. Regarding the overall postoperative complication rate, six studies reported Clavien–Dindo (CD) grade ≥ III, one study [23] reported CD grade ≥ II, and one study [19] did not report the overall complication rate. Therefore, these two studies [19, 23] were excluded from the meta‐analysis for the overall postoperative complication rate. However, for specific complications such as pulmonary complications and anastomotic leakage, the definitions varied among the included studies. To capture the maximum potential morbidity and provide a realistic overview of surgical risks, these specific complications were analyzed based on any reported occurrence in each study, regardless of the CD grade. In this analysis, pulmonary complications included pneumonia, pleural effusion, atelectasis, pleuritis, chylothorax, tracheal fistula, pulmonary fistula, and acute respiratory distress syndrome. The clinical indications for salvage esophagectomy, categorized as residual tumor immediately after dCRT or tumor relapse after clinical complete response, are summarized in Table 2. The surgical approaches (open thoracotomy, VATS, or RAMIE) and the extent of lymph node dissection (LND) performed at each institution are detailed in Table 3. These clinical characteristics were incorporated to evaluate the potential sources of heterogeneity among the included studies. The methodological quality of the included studies is listed in Table S1.

**TABLE 1:** Characteristics for included studies.

Author/year	No. of cases	Radiation dose (Gy)	Chemotherapy regimen	Overall postoperative complications rate (%)	Anastomotic leak rate (%)	Pulmonary complication rate (%)	Mortality rate (%)	R0 resection rate (%)	Survival rate (%)
Sohda M 2019^a^	21 scc	60	DCF 4 CF 8 NF 5 Doc 4	38 (≥ CD3)	29 (Any reported)	33 (Any reported)	14	67	3yOS:45.5% 5yOS:30.3%
Ohkura Y 2019	33 scc	≥ 50.4	DCF 5 CF 27 Others 1	33 (≥ CD3)	12 (≥ CD3)	15 (≥ CD3)	9	42	R1/2: 1yOS39.3% 5yOS 0%. R0: 1yOS 90.9% 5yOS 63.6%
Booka E 2020^a^	18 scc	60	CF	NA	39 (≥ CD2)	6 (≥ CD2)	0	78	1yOS 88.9% 2yOS 72.2% 5yOS 51.6%
Sugawara K 2020^a^	31 scc	50.4–65.4	CF 6 S1 + N 16 NF 9	29 (≥ CD3)	16 (Any reported)	35 (Any reported)	10	71	R1/2: 3yOS 0% R0: 3yOS 58.7%
Okamura A 2020	35 scc	50–70	CF 27 C 1 RT only 7	23 (≥ CD3)	14 (≥ CD2)	40 (≥ CD2)	9	54	1yOS 45.7% 2yOS 28.6% 5yOS 5.7%
Shiraishi O 2021^a^	37 scc	50–60	CF	32 (≥ CD3)^b^	12 (≥ CD2)^b^	35 (≥ CD2)^b^	9^b^	81	5yOS 26.0%
Defize IL 2021	23scc 1 adeno	50.4	CBDCA + PTX	80 (≥ CD2)	25 (≥ CD2)	54 (≥ CD2)	4	92	1yOS 83% 2yOS 51.0%
Tsuchiya N 2022^a^	9 scc	60	CF	33 (≥ CD3)	22 (≥ CD3)	33 (≥ CD3)	0	89	NA

Abbreviations: adeno, adenocarcinoma; C, CDDP; CBDCA, Carboplatin; CD, Clavien–Dindo grade; CF, CDDP, 5‐FU; DCF, docetaxel, CDDP, 5‐FU; Doc, docetaxel; N, nedaplatin; NA, not available; NF, nedaplatin, 5‐FU; OS, overall survival; PTX, paclitaxel; scc, squamous cell carcinoma.

^a^ Data of interest (salvage surgery cases) are extracted.

^b^ Without cases of R2 resection.

**TABLE 2:** Reasons for salvage esophagectomy (residual vs. relapse).

Author/year	No. of cases	Residual tumor (%)	Relapses (%)
Sohda M 2019	21 scc	9 (42.9%)	12 (57.1%)
Ohkura Y 2019	33 scc	21 (63.6%)	12 (36.4%)
Booka E 2020	18 scc	15 (83.3%)	3 (16.7%)
Sugawara K 2020	31 scc	31 (100%)	0 (0%)
Okamura A 2020	35 scc	34 (97.1%)	1 (2.9%)
Shiraishi O 2021	37 scc	37 (100%)^a^	0 (0%)^a^
Defize IL 2021	23scc 1 adeno	24 (100%)^a^	0 (0%)^a^
Tsuchiya N 2022	9 scc	9 (100%)	0 (0%)

Abbreviations: adeno, adenocarcinoma; scc, squamous cell carcinoma.

^a^ No explicit ‘relapse’ cases were documented; the subjects primarily consisted of planned surgery or residual disease cases determined by evaluations conducted after the completion of dCRT.

**TABLE 3:** Surgical approaches and extent of lymph node dissection (LND).

Author/year	No. of cases	Surgical approach (open vs. MIE)	Extent of LND
Sohda M 2019	21 scc	Not specified	Not specified
Ohkura Y 2019	33 scc	Open: 30 (90.9%)/VATS: 3 (9.1%)	Typical 2‐ or 3‐field LND, including prophylactic dissection
Booka E 2020	18 scc	Open Thoracotomy: 18 (100%)	Omitted typical or prophylactic LND. Limited to areas with suspected metastasis
Sugawara K 2020	31 scc	Open Thoracotomy: 31 (100%)	1‐ to 3‐field LND via right thoracoabdominal esophagectomy
Okamura A 2020	35 scc	Not specified (Decided by surgeons)	Not specified. Prophylactic LND may be omitted depending on residual tumor and PS
Shiraishi O 2021	37 scc	Open Thoracotomy: 37 (100%)	Standard 2‐ or 3‐field LND depending on disease progression
Defize IL 2021	23scc 1 adeno	RAMIE (Robot): 24 (100%)	Standard 2‐field LND
Tsuchiya N 2022	9 scc	Not specified	Standard 2‐ or 3‐field LND

Abbreviations: LND, lymph node dissection; MIE, minimally invasive esophagectomy; RAMIE, robot‐assisted minimally invasive esophagectomy; VATS, video‐assisted thoracoscopic surgery.

### 3.3 Outcomes and Meta‐Analysis

**FIGURE 2:**
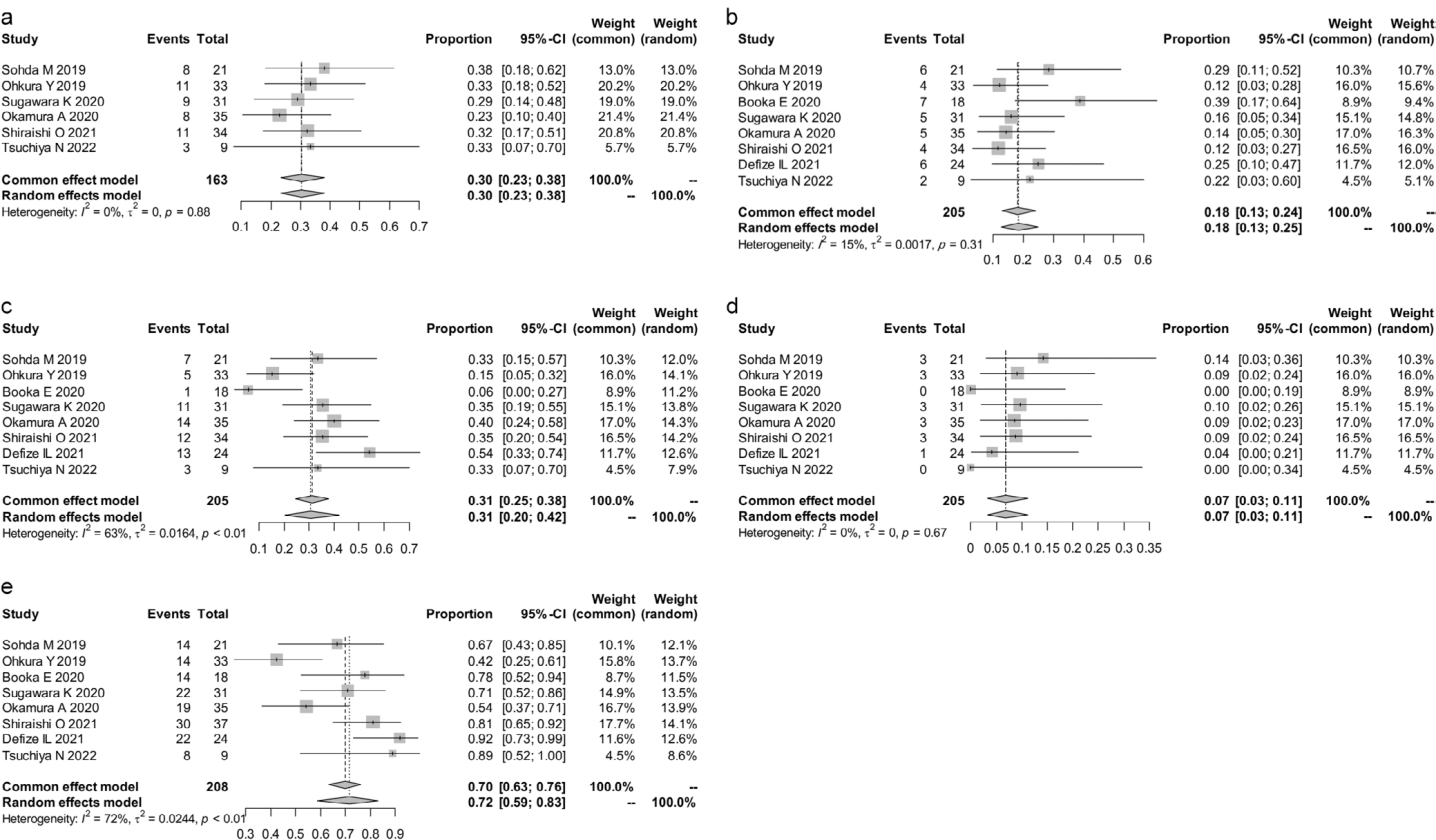
Forest plot of (a) overall postoperative complications rate, (b) anastomotic leak rate, (c) pulmonary complication rate, (d) mortality rate, and (e) R0 resection rate.

The pooled rate of endpoints were as follows: overall postoperative complications rate, 30% (95% CI: 23–38, *p* for heterogeneity = 0.88, *I* ^2^ = 0%, Figure 2a); anastomotic leak rate, 18% (95% CI: 13–25, *p* for heterogeneity = 0.31, *I* ^2^ = 15%, Figure 2b); pulmonary complication rate, 31% (95% CI: 20–42, *p* for heterogeneity < 0.01, *I* ^2^ = 63%, Figure 2c); mortality rate, 7% (95% CI: 3–11, *p* for heterogeneity = 0.67, *I* ^2^ = 0%, Figure 2d) and R0 resection rate, 72% (95% CI: 59–83, *p* for heterogeneity < 0.01, *I* ^2^ = 72%, Figure 2e).

### 3.4 Sensitivity Analysis

Significant statistical heterogeneity was observed in the rates of pulmonary complications and R0 resection. Sensitivity analyses were performed (Table S2). For the pulmonary complication rate, heterogeneity decreased significantly when the data from Booka et al. [19] were omitted (*I* ^2^ = 43%). This suggests that Booka et al. was a primary contributor to the observed heterogeneity in this outcome. For the R0 resection rate, heterogeneity remained high (*I* ^2^ > 64%) in all analyses except when the data from Ohkura et al. [18] were excluded, resulting in reduced heterogeneity (*I* ^2^ = 55%, *p* = 0.04). Furthermore, to address the clinical heterogeneity between residual and relapsed tumors, an additional sensitivity analysis was performed by excluding studies that included a relatively high proportion of relapse cases (Sohda et al. [17] and Ohkura et al. [18]). After excluding these two studies, the *I* ^2^ value for the R0 resection rate decreased from 72% to 60% (*p* = 0.03).

### 3.5 Assessment of Publication Bias

Figure S1a–e shows the Doi plots and LFK indexes associated with each outcome. According to the Doi plot, there was no asymmetry for overall postoperative complications (LFK index: −0.1), anastomotic leak (LFK index: −0.82), and mortality (LFK index: −0.82). On the other hand, there was major asymmetry for pulmonary complications (LFK index: −2.03) and R0 resection (LFK index: 2.36).

## 4 Discussion

This systematic review found that the overall postoperative complications rate was 30%; the anastomotic leak rate was 18%; the pulmonary complication rate was 31%; the mortality rate was 7%; and the R0 resection rate was 72% for salvage esophagectomy for T4 ESCC.

Short‐term outcomes of salvage esophagectomy have been reported in several studies [8, 25, 26], with reported morbidity rates of 50%–79% and mortality rates of 6%–22%. Miyata et al. reported that salvage esophagectomy, which included 37% T4 patients, was associated with an anastomotic leakage rate of 39%, a pulmonary complication rate of 30%, and a mortality rate of 12% [8]. Nishimura et al. reported an overall postoperative complication rate of 60%, anastomotic leakage rate of 22%, pneumonia rate of 9%, and a mortality rate of 15% for salvage esophagectomy cases; however, their study did not include T4 patients [25]. Watanabe et al. reported complication rates of 65.1% overall, anastomotic leakage rate of 15.9%, pulmonary complication rate of 36.5%, and a mortality rate of 7.9% for salvage esophagectomy, with 33% of patients being T4 [26]. Although most patients in these studies consisted of non‐T4 patients, we found similar morbidity and mortality rates for T4 patients. This suggests that the risk associated with salvage esophagectomy does not significantly differ based on pretreatment tumor depth. However, given the high complication rate identified in our analysis, this procedure should be recognized as a high‐risk intervention that requires intensive perioperative management in specialized centers.

R0 resection has been reported as an independent favorable prognostic factor for overall survival after salvage esophagectomy [26, 27]. Reported R0 resection rates ranged from 73% to 88% for salvage esophagectomy [8, 26, 27], which is similar to our pooled rate (72%). Although a formal meta‐analysis of survival was not feasible in our study, survival outcomes reported across included studies should also be considered along with R0 resection in evaluating the overall efficacy of salvage esophagectomy. Despite the high R0 resection rate, reported 5‐year overall survival rates ranged from 5.7% to 51.6% in our study. While the morbidity associated with salvage esophagectomy is high, its clinical significance is supported by the potential for achieving long‐term survival. In the studies included in this meta‐analysis, Booka et al. [19] reported a 5‐year OS rate of 51.6% in the salvage group, which was significantly higher than the 1.3% observed in the non‐surgical group. Other reports have also shown encouraging outcomes, such as a 5‐year OS rate of 30.3% reported by Sohda et al. [17]. Crucially, the long‐term prognosis appears to depend strictly on achieving R0 resection. Ohkura et al. [18] demonstrated that for patients who achieved R0 resection, the 5‐year OS rate reached 63.6%, whereas it was 0% for those with incomplete resection. Similarly, Sugawara et al. [20] showed that curative (R0) salvage surgery provided a 3‐year OS rate of 58.7%, which was comparable to the prognosis of patients achieving clinical complete response with dCRT alone. Our analysis underscores that while salvage surgery is a high‐risk procedure, it remains a meaningful curative option if R0 resection can be successfully achieved. This wide range of survival outcomes (5.7%–51.6%) despite relatively similar R0 rates suggests that patient selection criteria and surgical procedures, such as the extent of LND, were inconsistent across facilities.

Statistical heterogeneity was observed for pulmonary complications and the R0 resection rate. Sensitivity analysis indicated that the data from Booka et al. [19] contributed most to the heterogeneity observed for pulmonary complications (*I* ^2^ decreased from 63% to 43% when omitted). As detailed in Table 3, Booka et al. [20] intentionally omitted prophylactic LND to minimize the incidence of pneumonia (5.6%), whereas other centers performing standard LND reported higher rates. This statistical finding highlights a potential balance between oncological radicality and surgical safety in the context of salvage surgery. Sensitivity analysis also showed that the data from Sohda et al. [17] and Ohkura et al. [18] were the significant contributors to the heterogeneity for R0 resection. This heterogeneity may be attributed to the differing clinical backgrounds of the patients, specifically whether the surgery was performed for residual tumor immediately following dCRT or for tumor relapse after achieving a clinical complete response. Sohda et al. [17] and Ohkura et al. [18] reported substantially higher proportions of relapsed cases (57.1% and 36.4%, respectively) than other studies that primarily focused on residual disease. While the surgical intent remains curative in both scenarios, the pathological status and the degree of treatment‐induced fibrosis may differ significantly depending on the interval from dCRT. These factors can obscure surgical planes and complicate achieving R0 resection, leading to variability in outcomes across institutional series. Furthermore, the institutional criteria for cT4 diagnosis and resectability assessment were not standardized, which could introduce clinical heterogeneity. Our review found that, while diagnostic modalities were primarily enhanced CT, endoscopy, and endoscopic ultrasonography (EUS), interpretive thresholds for determining resectability varied significantly across centers. For instance, Ohkura et al. [18] adopted an aggressive surgical policy, justifying salvage surgery unless there was radiologically apparent destruction of the aortic or tracheal walls. In contrast, Okamura et al. [21] emphasized that the indication should be guided by the clinical response to dCRT, suggesting that surgery be reserved for cases where the residual tumor is downstaged to T2 or less. These differing surgical strategies regarding ‘resectability’ after dCRT likely contributed to the statistical variability observed in R0 resection rates across the included studies. Because of the limited number of studies available for analysis, we could not identify all potential factors contributing to heterogeneity. However, our analysis demonstrated that specific strategic differences, such as the extent of mediastinal lymph node dissection and the timing of surgery (residual versus relapse), are major drivers of the observed variability. These findings offer significant clinical insights and indicate that standardized criteria for resectability and surgical extent are essential to optimize outcomes in salvage esophagectomy.

The diversity of surgical approaches and the extent of LND across institutions represent significant sources of clinical heterogeneity in this meta‐analysis. While minimally invasive esophagectomy (MIE), including VATS and RAMIE, is generally expected to reduce respiratory complications, our findings suggest that the extent of mediastinal LND may play a more decisive role in pulmonary outcomes than the choice of surgical approach. For instance, Booka et al. reported a remarkably low pneumonia rate of 5.6% despite using open thoracotomy, which they attributed to the intentional omission of prophylactic LND to minimize morbidity in high‐risk salvage settings. Conversely, Defize et al. observed a higher rate of pulmonary complications (54%) associated with RAMIE and standard LND. This highlights a divergence in institutional surgical strategies: some centers prioritize oncological radicality through LND to improve long‐term prognosis, while others advocate limited LND to ensure perioperative safety. The high statistical heterogeneity in pulmonary complications likely reflects these varying strategic priorities rather than the approach's technological modality alone. Surgeons should therefore carefully balance the need for radical lymphadenectomy against the risk of severe respiratory morbidity when planning salvage surgery for cT4 disease.

The short‐term outcome of salvage esophagectomy for T4 ESCC demonstrated clinical feasibility, though it was associated with a high rate of severe complications in our study. Thus, several points should be interpreted with caution. First, most of the studies included in our analysis were published by Japanese institutions. This suggests that esophageal surgeons in Japan have relatively aggressive criteria for considering salvage surgery, even in cases that were initially deemed unresectable. Second, most of those institutions were recognized specialized esophageal surgery centers; thus, it is uncertain whether such results can be generalized to all centers. Third, careful consideration should also be given to the T4 diagnosis. Computed tomography and magnetic resonance imaging are widely used to detect local invasion of adjacent structures; however, a previous study reported T4 staging accuracies of 64% and 55%, respectively [28]. Additionally, variations exist among different observers in the clinical diagnosis of T4 ESCC [29]. Such discrepancies could lead to staging migration and impact outcome variability among institutions. Fourth, although salvage esophagectomy is widely performed for patients with stage I–IV esophageal cancer in real‐world clinical settings, it remains one of the most challenging procedures in surgical oncology for T4 disease. The Esophageal Cancer Practice Guidelines 2022 advises surgical resection for patients with initially unresectable, locally advanced ESCC that becomes resectable following definitive CRT, with careful consideration of safety, perioperative management, and the patient's informed consent [30].

This study has several limitations. First, all the studies included were case series. Thus, the influence of selection bias could not be excluded. Second, publication bias may have affected some of the outcomes of this study, as studies with negative results may have been underreported due to publishing difficulties. Such publication bias may lead to an overestimation of the outcomes. Third, we were unable to conduct a meta‐analysis of survival outcomes due to inconsistent reporting across studies. Fourth, the definition of postoperative mortality was not standardized across the included studies, with some studies reporting 30‐day or 90‐day mortality, in‐hospital mortality, or surgery‐ or treatment‐related mortality. While these varying criteria all aimed to capture deaths directly or indirectly attributable to the surgical intervention, this inconsistency may have affected the pooled mortality rate and should be considered when interpreting our findings. To further validate the efficacy and safety of T4 salvage esophagectomy in greater detail, additional prospective, randomized trials are required; however, these may be impractical due to the small number of eligible patients and the limited facilities available for performing the surgery. We are conducting a nationwide survey of patients who underwent salvage esophagectomy for cT4 thoracic ESCC at institutions accredited by the Japan Esophageal Society.

In conclusion, salvage esophagectomy for cT4 ESCC is a high‐risk procedure associated with significant morbidity, particularly a high anastomotic leak rate of 18%. Although R0 resection can offer a potential curative pathway, the generalizability of these results remains limited due to institutional variations in diagnostic criteria and surgical strategies. Further studies are needed to investigate the efficacy, safety, long‐term outcomes, and appropriate indications for this highly invasive but potentially beneficial procedure.

## Author Contributions

Systematic review and meta‐analysis: M.S. and K.K. Advice on article inclusion and exclusion: H.S. Statistical advice: K.N. Interpretation of data: M.S., K.K., and H.S. Drafting of manuscript: M.S. Critical revision: K.S. and H.S.

## Funding

The authors have nothing to report.

## Ethics Statement

All procedures were conducted in accordance with the ethical standards of the responsible institutional and national committees on human experimentation, as well as the Declaration of Helsinki (1964) and its subsequent amendments.

## Conflicts of Interest

Ken Shirabe is an editorial member of the Annals of Gastroenterological Surgery. The other authors declare that there are no conflicts of interest or funding associated with this manuscript.

## Supporting information


**Figure S1:** Doi plots and LFK indexes related to: (a) overall postoperative complications rate, (b) anastomotic leak rate, (c) pulmonary complication rate, (d) mortality rate, and (e) R0 resection rate.

**Table S1:** The methodologic quality of included studies.
**Table S2:** Sensitivity analysis.

## Data Availability

The data that support the findings of this study are available from the corresponding author upon reasonable request.
