# Supplementary material for: Systematic Review and Meta‐Analysis on the Efficacy and Safety of Salvage Esophagectomy for T4 Esophageal Squamous Cell Carcinoma
Source: Ann Gastroenterol Surg. 2026 May 5;10(5):1441–9. doi: 10.1002/ags3.70233 (PMC13394042; doi:10.1002/ags3.70233)
Supplement: Supplementary file 1 [file AGS3-10-1441-s001.docx]

**Supplementary Table 1. The methodologic quality of included studies**

|  | Sohda M 2019 | Ohkura Y 2019 | Booka E 2020 | Sugawara K 2020 | Okamura A 2020 | Shiraishi O 2021 | Defize IL 2021 | Tsuchiya N 2022 |
| --- | --- | --- | --- | --- | --- | --- | --- | --- |
| 1. **Were clear criteria reported for inclusion in the case series?** | Yes | Yes | Yes | Yes | Yes | Yes | Yes | Yes |
| 2. **Was the condition measured using a standard, reliable method for all participants included in the case series?** | Yes | Yes | Yes | Yes | Yes | Yes | Yes | Yes |
| 3. **Were valid methods used for identification of the condition for all participants included in the case series?** | Yes | Yes | Yes | Yes | Yes | Yes | Yes | Yes |
| 4. **Did the case series have consecutive inclusion of participants?** | Unclear | Yes | Yes | Yes | Yes | Yes | Yes | Yes |
| 5. **Did the case series have complete inclusion of participants?** | Unclear | Unclear | Unclear | Unclear | Unclear | Yes | Yes | Unclear |
| 6. **Was there clear reporting of the demographics of the participants in the study?** | Yes | Yes | Yes | Yes | Yes | Yes | Yes | Yes |
| 7. **Was there clear reporting of clinical information of the participants?** | Yes | Yes | Yes | Yes | Yes | Yes | Yes | Yes |
| 8. **Were the outcomes or follow-up results of cases clearly reported?** | Yes | Yes | Yes | Yes | Yes | Yes | Yes | Yes |
| 9. **Was there clear reporting of the presenting site(s’)/clinic(s’) demographic information?** | Yes | Yes | Yes | Yes | Yes | Yes | Yes | Yes |
| 10. **Was statistical analysis appropriate?** | Unclear | Yes | Yes | Yes | Yes | Yes | Yes | Yes |

**Supplementary Table 2. Sensitivity Analysis**

| Study omitted | Pooled effect sizes (%) | 95% Cl (%) | Heterogeneity |  |
| --- | --- | --- | --- | --- |
|  |  |  | ***I*^2^ (%)** | **p-value** |
| Pulmonary complication rate |  |  |  |  |
| Sohda M 2019 | 30 | 19–43 | 68 | <0.01 |
| Ohkura Y 2019 | 34 | 23–45 | 55 | 0.04 |
| Booka E 2020 | 34 | 25–44 | 43 | 0.10 |
| Sugawara K 2020 | 30 | 18–43 | 68 | <0.01 |
| Okamura A 2020 | 29 | 17–42 | 66 | <0.01 |
| Shiraishi O 2021 | 30 | 18–43 | 68 | <0.01 |
| Defize IL 2021 | 28 | 18–38 | 54 | 0.04 |
| Tsuchiya N 2022 | 30 | 19–43 | 68 | <0.01 |
|  |  |  |  |  |
|  |  |  |  |  |
| R0 resection rate |  |  |  |  |
| Sohda M 2019 | 72 | 58–85 | 76 | <0.01 |
| Ohkura Y 2019 | 76 | 65–85 | 55 | 0.04 |
| Booka E 2020 | 71 | 56–84 | 75 | <0.01 |
| Sugawara K 2020 | 72 | 57–85 | 76 | <0.01 |
| Okamura A 2020 | 74 | 61–86 | 71 | <0.01 |
| Shiraishi O 2021 | 70 | 55–83 | 72 | <0.01 |
| Defize IL 2021 | 68 | 55–79 | 64 | 0.01 |
| Tsuchiya N 2022 | 70 | 56–82 | 74 | <0.01 |
| Sohda M 2019 and Ohkura Y 2019 | 77 | 65-88 | 60 | 0.03 |
