# Supplementary figures and images for: Systematic Review and Meta‐Analysis on the Efficacy and Safety of Salvage Esophagectomy for T4 Esophageal Squamous Cell Carcinoma
Source: Ann Gastroenterol Surg. 2026 May 5:10.1002/ags3.70233. Online ahead of print. doi: 10.1002/ags3.70233 (PMC13394042; doi:10.1002/ags3.70233)

## Slide 1
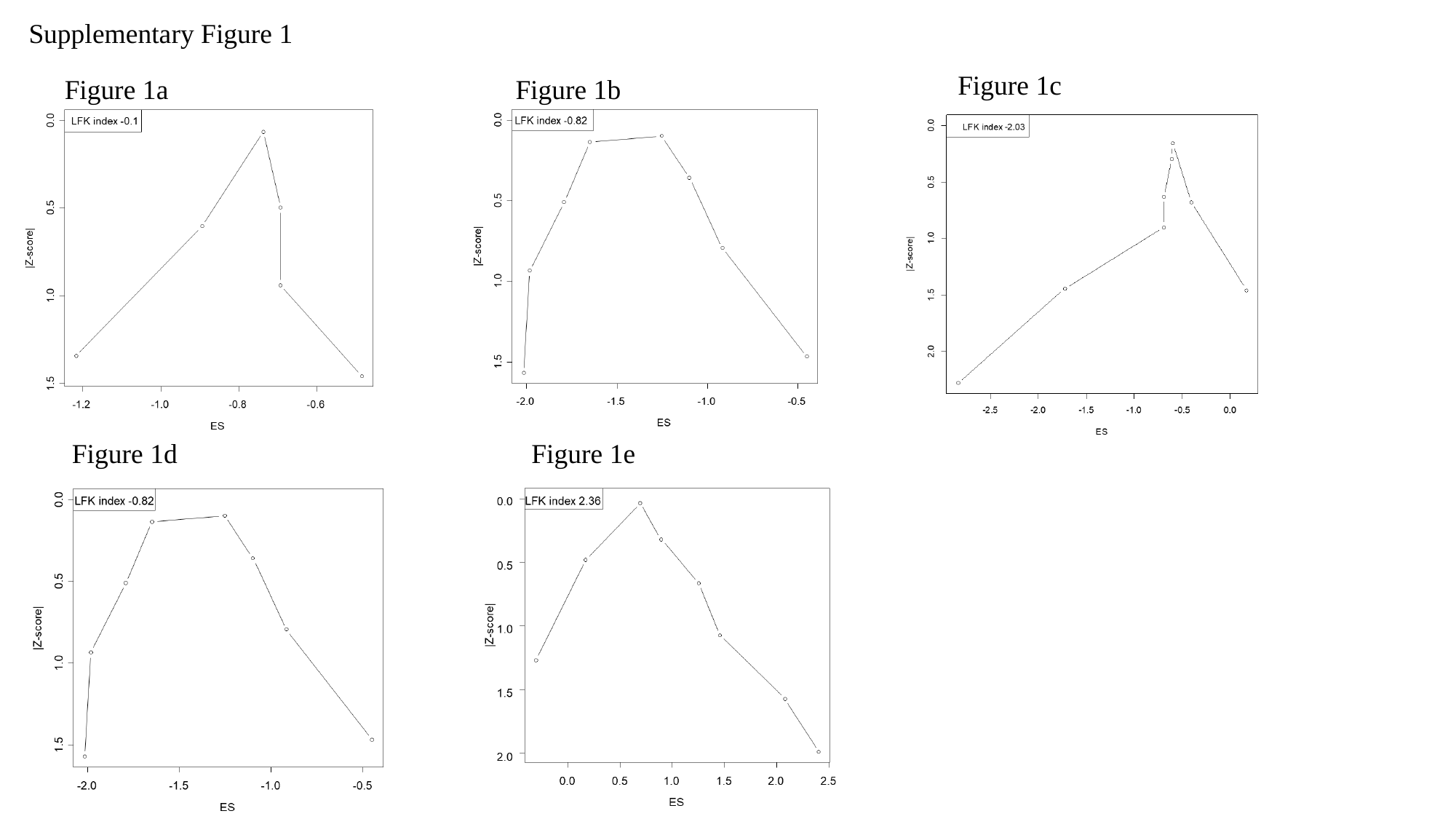

Supplementary Figure 1
 Figure 1c
 Figure 1a
 Figure 1b
 Figure 1d
 Figure 1e

Supplement: Supplementary file 1 — Figure S1: Doi plots and LFK indexes related to: (a) overall postoperative complications rate, (b) anastomotic leak rate, (c) pulmonary complication rate, (d) mortality rate, and (e) R0 resection rate. [file AGS3-9999-0-s002.pptx]
